# Association between Genetic Variants in DNA Double-Strand Break Repair Pathways and Risk of Radiation Therapy-Induced Pneumonitis and Esophagitis in Non-Small Cell Lung Cancer

**DOI:** 10.3390/cancers8020023

**Published:** 2016-02-18

**Authors:** Lina Zhao, Xia Pu, Yuanqing Ye, Charles Lu, Joe Y. Chang, Xifeng Wu

**Affiliations:** 1 Department of Epidemiology, The University of Texas MD Anderson Cancer Center, 1515 Holcombe Blvd, Houston, TX 77030, USA; zhaolinazln@outlook.com (L.Z.); ipuxia@gmail.com (X.P.); yqye@mdanderson.org (Y.Y.); 2 Department of Radiation Oncology, Xijing Hospital, Fourth Military Medical University, Xi’an 710032, China; 3 Department of Thoracic/Head and Neck Medical Oncology, The University of Texas MD Anderson Cancer Center, 1515 Holcombe Blvd, Houston, TX 77030, USA; jychang@mdanderson.org; 4 Departement of Radiation Oncology, The University of Texas MD Anderson Cancer Center, 1515 Holcombe Blvd, Houston, TX 77030, USA; clu@mdanderson.org

**Keywords:** NSCLC, radiation pneumonitis and esophagitis, DNA double-stranded break repair pathway, single nucleotide polymorphism

## Abstract

Radiation therapy (RT)-induced pneumonitis and esophagitis are commonly developed side effects in non-small cell lung cancer (NSCLC) patients treated with definitive RT. Identifying patients who are at increased risk for these toxicities would help to maximize treatment efficacy while minimizing toxicities. Here, we systematically investigated single nucleotide polymorphisms (SNPs) within double-strand break (DSB) repair pathway as potential predictive markers for radiation-induced esophagitis and pneumonitis. We genotyped 440 SNPs from 45 genes in DSB repair pathways in 250 stage I–III NSCLC patients who received definitive radiation or chemoradiation therapy, followed by internal validation in 170 additional patients. We found that 11 SNPs for esophagitis and 8 SNPs for pneumonitis showed consistent effects between discovery and validation populations (same direction of OR and reached significance in meta-analysis). Among them, rs7165790 in the *BLM* gene was significantly associated with decreased risk of esophagitis in both discovery (OR = 0.59, 95% CI: 0.37–0.97, *p* = 0.037) and validation subgroups (OR = 0.45, 95% CI: 0.22–0.94, *p* = 0.032). A strong cumulative effect was observed for the top SNPs, and gene-based tests revealed 12 genes significantly associated with esophagitis or pneumonitis. Our results support the notion that genetic variations within DSB repair pathway could influence the risk of developing toxicities following definitive RT in NSCLC.

## 1. Introduction

Radiation therapy (RT) is the primary care for locally advanced non-small cell lung cancer (NSCLC). However, while resulting in tumor shrinkage, RT also leads to damage of surrounding normal tissues and caused normal tissue toxicities [1]. Commonly occurred RT-induced toxicities have limited the planed dose and therefore compromised the efficiency of local and/or regional control and negatively influence patients’ prognosis. Accurate prediction before treatment will therefore enable personalized dosimetric design, and maximize treatment effect while minimizing potential toxicities. Currently, the risk of RT-induced toxicities were assessed based on clinical-based factors, including physical characteristics of the radiation beam; treatment dose, fractionation and time; volume and type of normal tissue receiving radiation; radiosensitizer usage; and comorbidities [2]. However, large variation exists in patients with similar clinical parameters. It suggests a potential role of host genetic factors in determining an individual’s response to RT and therefore susceptibility of developing RT-induced toxicities [3].

Generally, DNA double-strand breaks (DSBs) are the major genotoxic lesions induced by ionization radiation. There are two distinct and complementary pathways for DSB repair, namely, homologous recombination (HR) and non-homologous end joining (NHEJ). DSBs repairing play a key role in maintaining genomic stability and integrity after RT [4]. It has been reported that deficiencies in DSB repair genes were associated with high incidence of chromosome aberrations and increased tumor radiation sensitivity [5,6,7,8]. Therefore, it is reasonable to presume that variants in DSB repair pathway genes could modify the radiosensitivity as well as clinical outcomes after RT.

Several studies have reported the association between SNPs in several DSB repair pathway genes and RT-induced esophagitis and pneumonitis, such as *BRCA1* [9], *ATM* [10,11], *P53* [10], *RAD51* [4] and *LIG4* [12]. However, these studies mainly used single gene-based approach without validation of their findings. We have previously used pathway-based approaches to identify genetic variations in inflammation pathway genes as predictors of radiation-induced toxicities in NSCLC patients [13], which provided more coverage compared to single-gene-based approaches. In this study, to the best of our knowledge, we, for the first time, utilized a pathway-based approach to investigate genetic variations within DSB pathway genes in a relatively large, well-characterized population and analyzed their role in developing esophagitis or pneumonitis following definitive RT with a validation step. Our goal is to identify potential DSB-related biomarkers which will be used to facilitate personalized dosage design.

## 2. Materials and Methods

### 2.1. Study Population and Data Collection

Study patients were newly diagnosed and histologically confirmed stage I–III NSCLC patients recruited between September 1995 and February 2008. All these patients had received chemoradiation therapy or definitive thoracic radiation at The University of Texas MD Anderson Cancer Center. Tumor staging was defined based on the 6th edition of American Joint Committee on Cancer (AJCC) staging. A structured questionnaire was used to collect epidemiological data during an in-person interview conducted by a well-trained staff interviewer. Clinical as well as follow-up information was abstracted from medical records. Pretreatment performance status was defined based on the Eastern Cooperative Oncology Group scale. Definitions of radiation-induced pneumonitis and esophagitis have been previously reported [13]. In brief, symptomatic pneumonitis was defined as clinical presentation of patients with respiratory complaints during and after radiation treatment, including dyspnea and chest pain in the setting of absence of evidence for infection. Similarly, for esophagitis, symptomatic complaints related to swallowing including dysphagia, odynophagia or chest discomfort at baseline during and after radiation treatment were included in the definition. Severity of esophagitis or pneumonitis was scored by the clinical physicians according to the National Cancer Institute Common Terminology Criteria for Adverse Events (version 3.0) guidelines [14]. For pneumonitis and esophagitis, toxicity was scored as grade 1 (asymptomatic: radiographic or endoscopic findings only), grade 2 (moderate symptoms: altered breathing or dietary habits requiring medical intervention), grade 3 (severe symptoms: oxygen indicated; unable to aliment orally), grade 4 (life-threatening: ventilator support indicated), or grade 5 (death). Final determination of radiation toxicities was determined by the considerations of patient clinical findings made by the treating radiation oncologist. Consistent with prior studies [10,13,15,16], occurrence of grade ≥ 2 toxicities was considered as an event in this study since grade 1 pneumonitis or esophagitis is clinically asymptomatic and does not require medical intervention. A blood sample was drawn from each participant for subsequent analysis. All patients signed an informed consent form, and the study was approved by the Institutional Review Board of MD Anderson.

### 2.2. SNP Selection and Genotyping

SNPs were genotyped using a custom Illumina iSelect Infinium II genotyping platform (Illumina, San Diego, CA) containing 9645 SNPs from 998 genes. The details for the chip design, including SNP and gene selection methods, have been described previously [17]. Briefly, tagging SNPs for each gene were selected from within a 10-kb flanking region using CEU data from the HapMap Project [18], based on the NCBI B36 assembly and dbSNP b126 by using the Tagger Pairwise method (r^2^ > 0.8 and minor allele frequency (MAF) ≥ 0.05) as reported previously [19]. SNPs located in the coding (synonymous and non-synonymous SNPs), in regulatory regions (promoter, 3′ or 5′-untranslated regions (UTRs)) or at splicing sites were also selected, and a SNP is also included if it has previously been reported as a potentially functional SNP with an MAF greater than 1% in the Caucasian population.

A total of 440 SNPs in 45 candidate genes in DNA DSB repair pathway were included in this panel (Supplementary Table S1). Genomic DNA was extracted from peripheral blood lymphocytes using the QIAamp DNA extraction kit (Qiagen, Valencia, CA, USA). Genotyping was performed according to the standard Infinium II assay protocol, and any DNA samples or SNPs with a call rate (percentage of data available for all SNPs or samples) <95% were excluded from further analysis. For the validation phase, the genotyping was performed using HumanHap300k and 370k BeadChip (Illumina, San Diego, CA, USA). Quality control rules were similar to those used in the discovery phase—only SNPs and samples with a genotyping or sample call rate >95% were included in the analysis.

### 2.3. Statistical Analysis

A Chi-square test and a Student’s t-test were used to assess the distribution of covariates between patients with or without an event. Multivariate logistic regression was used to estimate the main effect of single SNP on risk of developing toxicities adjusted for age, sex, pack year, clinical stage, performance status, chemoradiotherapy, radiation treatment type, forced expiratory volume in the first second (FEV1), carbon monoxide diffusing capacity (DLCO) percentage, planning tumor volume (PTV), mean dose (mean esophagus dose for esophagitis and mean lung dose for pneumonitis). For each SNP dominant, recessive and additive models were analyzed, and only the best-fitting model were reported. In order to expand the coverage of the validation panel, we also included proxy SNPs that are in high linkage disequilibrium (LD > 80%) with the original genotyped SNP from the discovery phase and analyzed in the validation population. The proxy SNPs were identified using SNAP [20] based on LD information calculated using phased genotype data from the International HapMap Project and the 1000 Genomes Project.

If the proxy SNPs showed a similar effect to the original SNP in the discovery phase, meta-analysis was performed to summarize the effects from discovery and validation populations. Heterogeneity was estimated using χ^2^-based Q-statistics. A fixed effect model was used when heterogeneity was absent (*p* for heterogeneity <P-het> >0.05). An unfavorable genotype (UFG) was defined as the allele associated with an increased risk of developing toxicities. A joint analysis all UFGs for each patient and risk of esophagitis or pneumonitis was conducted with subjects stratified by level of risk in tertiles. All statistical analyses were two-sided. The analysis described above was performed using STATA software (version 10, STATA Corp, College Station, TX, USA).

HaploReg [21] was used for functional annotations of candidate SNPs. PolyPhen-2 [22], SNPeffect [23], SIFT [24] and SNPs3D [25] were used to predict the function of missense variant on protein function. VEGAS was used to perform gene-based tests, which produces a gene-based test statistic based on a simulation to calculate an empirical gene-based *p*-value [26].

## 3. Results

### 3.1. Characteristics of Patients

A total of 420 (250 in discovery, 170 in validation) Stage I–III NSCLC patients were included in this analysis. Table 1 summarizes the baseline clinical characteristics of patients in the discovery and validation phases, respectively. No statistically significant difference was observed between patients in the discovery and validation phases in terms of age, gender, smoking pack year, performance status, pulmonary function, PTV volume, mean esophagus dose or proportion of radiation-induced esophagitis. Patients in the validation phase had slightly lower mean lung dose (16 *vs*. 18 Gy), were less likely to receive concurrent chemoradiation therapy (35% *vs*. 74%), more patients treated by IMRT, and less treated by 3D-CRT technique (41% *vs*. 23%) compared to the discovery phase.

### 3.2. Association between Individual SNPs and Esophagitis Risk

A total of 440 SNPs were included in the discovery phase. Fifty SNPs in 20 genes were significantly associated with esophagitis (*p* value <0.05, Supplementary Table S2). Among them, genotyping data was available for 30 SNPs (15 original and 15 proxy SNPs (Supplementary Table S3). Among them, 11 SNPs showed the same trend of effects between discovery and validation populations and reached statistical significance in the combined meta-analysis (Table 2). Some of the SNPs profiled in the discovery were not found in the GWAS panel during validation; hence, proxy SNPs were selected and analyzed. Rs7165790 and its proxy SNP rs7175811 are intronic SNPs in *BLM* and showed consistent effect and significant association in the above two populations. The two SNPs were associated with a significantly decreased risk of esophagitis under the additive model (discovery: OR = 0.59, 95% CI: 0.37–0.97, *p* = 0.037; validation: OR = 0.45, 95% CI: 0.22–0.94, *p* = 0.032, respectively). Meta-analysis of the association of rs7165790 and rs7175811 with radiation esophagitis under the fixed effects model showed a *p* value of 0.003 (OR = 0.54, 95% CI: 0.36–0.82, *p* for heterogeneity = 0.462).

### 3.3. Association between Individual SNPs and Pneumonitis Risk

31 SNPs in 17 genes were found to be associated with pneumonitis (Supplementary Table S4). Among the 31 SNPs, genotyping data was available for 23 SNPs (7 original SNPs and 16 proxy) in the validation phase (Supplementary Table S5). SNPs (r^2^ > 0.8) were genotyped by GWAS. In total, 8 SNPs showed the same trend effect between the discovery and validation populations, and all of them reached significance by joint meta-analysis (Table 3).

### 3.4. Cumulative Effects for Esophagitis or Pneumonitis Risk

To analyze the cumulative effect of identified SNPs on radiation toxicities, we performed UFG analysis on both outcomes using the discovery population (Table 4). SNPs showing consistent effects between discovery and validation populations and reaching statistical significance in the meta-analysis were included in the UFG analysis. Significant dose-respond effects were identified for both esophagitis and pneumonitis risks as the number of unfavorable genotypes increased (*p* trend for the individual UFG was 2.59 × 10^−7^ and 6.34 × 10^−8^ respectively). The risk of developing esophagitis for patients carrying 6 to 7 unfavorable genotypes was increased 3.63-fold compared to those less than 6 risk genotypes (*p* = 0.003). The risk was dramatically increased 113.12-fold when 8 to 10 unfavorable genotypes were applied compared to patients with 0 to 5 UFGs (*p* = 5.46 × 10^−6^). The increased risk of developing pneumonitis for patients carrying 3 to 5 unfavorable genotypes was 8.65-fold higher compared to patients with 0 to 2 UFGs (*p* = 2 × 10^−4^). The risk was dramatically increased 73-fold when 6–8 unfavorable genotypes were applied (6.70 × 10^−6^).

### 3.5. In Silico SNP Function Prediction

To further investigate the identified variants and explore potential mechanisms, we used bioinformatics tools to evaluate their effects on protein structure and function (Table 5). HaploReg identified that six SNPs were located in enhancer histone marks (rs2270132, rs1822744, rs401549, rs16944739, rs3735461 and rs11571468). rs3735461, which was associated with an increased risk of pneumonitis, was predicted to occur in protein-binding regions and also in promoter histone mark sites. 16 SNPs were predicted to alter regulatory binding motifs, and 7 SNPs (rs2270132, rs10514249, rs1051772, rs16944739, rs3735461, rs11571468 and rs917029) were located in DNase sites.

Homozygous variant GG genotype of rs1799966 in *BRCA1* was a missense variant that was associated with increased risk of radiation esophagitis. The polymorphism results in amino acid change from a polar and uncharged serine to a nonpolar uncharged glycine. SIFT (SIFT score: 0.02), SNPeffect and SNPs3D analysis indicated this amino acid change may have a deleterious effect on protein function. Another missense SNP, *BRIP1*:rs4986764, which was associated with increased risk of radiation pneumonitis. This variant results in amino acid change from a polar and uncharged serine residue to a nonpolar uncharged proline. SNPeffect analysis indicated this amino acid substitution may have a deleterious effect on protein function.

### 3.6. Gene-Based Analysis

We further used VEGAS to perform gene-based tests to summarize the effect of SNPs within a single gene on toxicities (Table 6). In total, 7 genes (*EXO1*, *RPA1*, *MDC1*, *BLM*, *RAD54L*, *BRCA1*, *PRKDC*) were significantly associated with radiation-induced esophagitis, among which *EXO1* had the smallest *p* value (*p* = 0.005). 6 genes (*RAD54L*, *MUS81*, *RAG1*, *RAG2*, *EME1*, *ATM*) were found to be significantly associated with radiation-induced pneumonitis, among which *RAD54L* had the smallest *p* value (*p* = 0.033).

## 4. Discussion

RT destroys tumor cells mainly by damaging their DNA, especially double-strand DNA. However, such an effect also occurs in normal cells. An individual’s capacity for repairing DNA damage may determine the presence and extent of radiation toxicities. It has been known for years that genetic variations in DNA repair genes might regulate patients’ sensitivity to RT [1]. In this study, we systematically investigated genetic variation within DSB repair pathway as potential predictive biomarkers for radiation-induced esophagitis and pneumonitis in NSCLC patients treated with definitive radiation or concurrent chemoradiation. To control for false discovery, a validation step was also included. We found that an intronic SNP *BLM*:rs7165790 was significantly associated with a decreased risk of esophagitis in both the discovery and validation populations. A strong cumulative effect was observed for these SNPs on radiation toxicities.

The most significant SNP for esophagitis was an intronic variant *BLM*:rs7165790. Bloom syndrome (BS) gene product, *BLM*, encodes the protein RecQL3 helicase, an enzyme that restores malfunctioning replication forks during DNA replication. The RecQ helicase BLM has been also shown to be involved in single-strand DNA resection at the initial stages of homologous recombination [27,28]. Grabarz *et al.* found that *BLM* is an essential factor involved in DSB repair initiation and is essential for the maintenance of genome stability [29]. Mutations in *BLM* could lead to Bloom syndrome, which is an autosomal-recessive genetic disorder that is associated with increased levels of spontaneous sister-chromatid exchanges (SCEs), genome instability, as well as elevated cancer susceptibility [30]. Broberg *et al.* performed a case-control study to indicate that a variant allele of rs2532105 in BLM showed increased risk for breast cancer [31]. In our study, *BLM*:rs7165790 was the only SNP validated to be associated with decreased risk of developing esophagitis. Gene-based test also showed that *BLM* was among the most significant genes associated with risk of esophagitis. All these results support that this gene plays an important role in radiation-induced esophagitis. Although located in the intron region where its function was not obvious, rs7165790 was identified as borderline significant in expression quantitative trait locus (eQTL) relationship with *BLM* (*p* = 0.0644). It is possible that this SNP, or other SNPs tagged by it, could contribute to the alteration of *BLM* expression, which warrants further investigation.

For the radiation-induced pneumonitis, the top SNP was synonymous with SNP rs1051772 in *TOPBP1*, which was significantly associated with decreased risk of radiation-induced pneumonitis and showed the same trend of effects between discovery and validation populations and reached statistical significance in the combined meta-analysis. Increasing evidence has indicated that *TOPBP1* participated in DNA replication checkpoint control and played important roles in maintaining genomic stability [32]. SNPs in *TOPBP1* were also associated with some cancer risks [10,11]. The mechanisms underlying radiation-induced toxicities need to be further explored. *MUS81* was the top gene that was significantly associated with radiation-induced pneumonitis by gene-based analysis. 3 SNPs (rs13817, rs558114 and rs635375) in *MUS81* were shown to be significantly related to radiation-induced pneumonitis in the discovery group only, and the exact function is still unclear. The N-terminus was also proposed to contain a BLM-interacting domain [33], and whether these two genes interact with each other in mediating radiation toxicities needs to be further studied.

Previous studies have reported the association of *ATM* polymorphisms with radiation-induced esophagitis [34,35,36]. In our study, we did not find a similar association, although 10 genetic variants tagging the *ATM* gene were included in the analysis. Since most of the prior reports are candidate gene studies genotyping a limited number of SNPs in the Asian population while our study is pathway-based in the non-Hispanic white population, differences in study design and demographic group may affect the final results.

Interestingly, we did not find overlapping SNPs between esophagitis and pneumonitis, suggesting that different biological mechanisms or responses to chemoradiation-induced damage may play a role in the development of these toxicities. This is supported by our finding that compared to radiation-only treatment, concurrent chemoradiation treatment did not seem to affect the distribution of patients with pneumonitis but significantly increased the incidence of esophagitis (Supplementary Table S6). However, since this study only focused on genetic variants in the DSB pathway, we could not rule out that other pathways, such as inflammation, might share common susceptibility factors for esophagitis and pneumonitis. Future whole-genome profiles of genetic variation associated with these adverse events are necessary to find the most significant and/or common genetic factors for clinical application. 

Our study has several advantages. First, other than single genes, we systematically investigated the effects of genetic variations (440 SNPs) within major genes in DNA-DSB repair pathway (44 genes). Second, we performed the first comprehensive dosimetric and clinical data collection to enable this pathway-based analysis. Moreover, to reduce the potential false positive findings, we adopted a two-phase screening and validation approach in the analysis.

There were, however, some limitations to this study. First, the sample size was relatively small due to the fact that radiation therapy is mostly used in late-stage patients in our population, while the majority of patients were treated initially by surgery or by a combination of surgery and chemotherapy. However, the sample size is relatively large compared to other studies of this kind [37]. Second, although we included an internal validation group, the possibility of false positives still exists. Future external independent validation should be included to further validate our findings.

In summary, this is the first pathway-based study for association between single nucleotide polymorphisms in DNA DSB repair pathway genes and risk of radiation-induced pneumonitis and esophagitis for NSCLC Patients. Our results provide strong support for the claim that DSB pathway-related genetic variations serve as a potential biomarker to predict radiation toxicities and further guide the RT for NSCLC patients. With further investigations, we can test the predictive value by combining these significant SNPs with some commonly used variables in clinics that may be also related to radiation toxicities.

## Figures and Tables

**Table 1:** Patient characteristics.

Variable	Discovery Phase, *n* (%)	Validation Phase, *n* (%)
**Age, mean (SD)**	62.9 (10.1)	63.9 ( 9.9)
**Pack year, mean (SD)**	52.2 (28.5)	52.6 (28.5)
**DLCO percentage, mean (SD)**	66.6 (20.8)	65.9 (19.8)
**FEV1 percentage, mean (SD)**	71.5 (18.1)	66.7 (21.1)
**PTV volume (cm^3^) ,mean (SD)**	729.9 (491.4)	655.5 (384.8)
**Mean esophagus dose, mean (SD)**	29.8 (13.1)	27.5 (10.8)
**Mean lung dose, mean (SD)**	18.0 (8.3)	16.0 ( 5.6)
**Median total dose (range)**	63 (34.8–126)	60 (10.9–83.8)
**Sex**		
Male	129 (52)	95 (56)
Female	121 (48)	75 (44)
**Clinical stage**		
I	43 (17)	2 (1)
II	40 (16)	9 (5)
IIIA	92 (37)	113 (66)
IIIB	75 (30)	46 (27)
**Histology**		
Adenocarcinoma	90 (36)	68 (40)
Large cell carcinoma	13 (5.2)	8 (4.7)
Squamous cell carcinoma	84 (33.6)	63 (37)
Other	63 (25.2)	31 (18.2)
**Performance status**		
0	72 (29)	41 (24)
1	117 (47)	73 (43)
2–4	23 (9)	22 (13)
**Treatment modality**		
Radiation	65 (26)	110 (65)
Concurrent chemoradiation	185 (74)	60 (35)
Platinum-based only	77 (41.6)	21 (35)
Platinum plus taxane	108 (58.4)	39 (65)
**Radiation Type**		
2D	74 (30)	51 (30)
3D	105 (42)	40 (24)
IMRT	58 (23)	69 (41)
Proton	13 (5)	10 (6)
**Esophagitis**		
No	106 (43)	104 (62)
Yes	143 (57)	65 (38)
**Pneumonitis**		
No	147 (63)	91 (61)
Yes	87 (37)	58 (39)
**Total**	250	170

FEV1: forced expiratory volume in the first second; DLCO: carbon monoxide diffusing capacity; NOS: not otherwise specified; PTV: planning tumor volume; IMRT: intensity modulated radiotherapy, Numbers may not add to total due to missing clinical information.

**Table 2:** Selected SNPs associated with radiation esophagitis.

SNP	Gene	Allele	Model	Discovery		Validation		Meta		
				OR ^#^ (95% CI)	*p* Value	OR ^#^ (95% CI)	*p* value	OR (95% CI)	*p* Value	P-het
rs7165790 ^&^	BLM	A > G	add	0.59 (0.37–0.97)	0.037	0.45 (0.22–0.94)	0.032	0.54 (0.36–0.82)	0.003	0.462
rs8176257 ^	BRCA1	C > A	rec	5.42 (1.47–20.03)	0.011	3.18 (0.75–13.49)	0.116	4.27 (1.62–11.24)	0.003	0.592
rs2270132 ^	BLM	A > C	dom	2.59 (1.27–5.26)	0.009	1.75 (0.65–4.74)	0.268	2.27 (1.27–4.04)	0.005	0.533
rs12516 *	BRCA1	G > A	rec	3.89 (1.12–13.54)	0.032	3.31 (0.79–13.79)	0.100	3.63 (1.42–9.28)	0.007	0.866
rs1799966 ^	BRCA1	A > G	rec	3.89 (1.12–13.54)	0.032	3.31 (0.79–13.79)	0.100	3.63 (1.42–9.28)	0.007	0.866
rs4873772 *	PRKDC	G > A	rec	7.17 (1.77–29.05)	0.006	1.28 (0.30–5.46)	0.743	3.06 (0.56–16.62)	0.027	0.094
rs1822744 *	TOPBP1	A > G	add	1.86 (1.1–3.13)	0.021	1.24 (0.69–2.24)	0.473	1.55 (1.05–2.3)	0.027	0.317
rs11078671 *	RPA1	C > A	rec	4.17 (1.19–14.61)	0.026	1.69 (0.5–5.71)	0.400	2.62 (1.08–6.36)	0.031	0.311
rs401549 ^	BLM	A > G	add	1.91 (1.14–3.2)	0.013	1.08 (0.54–2.17)	0.821	1.51 (0.87–2.62)	0.034	0.197
rs1776139 *	EXO1	A > C	dom	0.45 (0.21–0.98)	0.044	0.69 (0.28–1.68)	0.414	0.54 (0.3–0.97)	0.040	0.479
rs10514249 *	XRCC4	A > G	rec	0.39 (0.17–0.89)	0.024	0.86 (0.27–2.69)	0.792	0.52 (0.25–1.09)	0.047	0.273

**^#^** adjusted for age, sex, pack year, clinical stage, performance status, concurrent chemoradiotherapy, radiation treatment type, FEV1 percentage, DLCO percentage, PTV volume, mean esophagus dose and mean lung dose; * Use proxy SNP in validation; ^ Re-genotyped in GWAS population using original SNP found in the discovery phase; ^&^ Use proxy SNP rs7175811 in validation stage from GWAS data; Abbreviations: Dom, dominant; Rec, recessive; Add, additive; P-het, P for heterogeneity.

**Table 3:** Eight DNA double-strand break repair pathway related SNPs with same trend effects on radiation pneumonitis across two analytical phases.

				Discovery		Validation		Meta		
SNP	Gene	Allele	Model	OR ^#^ (95% CI)	*p* value	OR ^#^ (95% CI)	*p* value	OR (95% CI)	*p* value	P-het
rs1051772 ^	TOPBP1	A > G	dom	0.27 (0.11–0.65)	0.004	0.41 (0.13–1.27)	0.122	0.32 (0.16–0.63)	0.001	0.564
rs16944739 *	BLM	G > A	rec	3.11 (1.13–8.56)	0.028	6.24 (0.68–57.4)	0.106	3.51 (1.40–8.81)	0.008	0.576
rs3735461 ^	RPA3	A > G	dom	2.90 (1.39–6.04)	0.005	1.41 (0.61–3.24)	0.418	2.11 (1.22–3.66)	0.008	0.204
rs963248 *	XRCC4	A > G	dom	1.92 (1.01–3.63)	0.047	2.02 (0.84–4.83)	0.115	1.95 (1.16–3.27)	0.011	0.925
rs3760412 *	EME1	A > G	dom	0.42 (0.22–0.79)	0.008	0.84 (0.39–1.79)	0.651	0.56 (0.34–0.91)	0.019	0.170
rs11571468 ^	RAD52	G > A	dom	0.35 (0.13–0.95)	0.040	0.55 (0.17–1.73)	0.305	0.43 (0.20–0.90)	0.026	0.566
rs4986764 *	BRIP1	G > A	rec	2.42 (1.08–5.45)	0.032	1.53 (0.52–4.56)	0.443	2.06 (1.07–3.95)	0.030	0.509
rs917029 *	EME1	A > G	rec	2.57 (1.09–6.08)	0.031	1.53 (0.50–4.65)	0.456	2.12 (1.07–4.18)	0.030	0.467

**^#^** adjusted for age, sex, pack year, clinical stage, performance status, concurrent chemoradiotherapy, radiation treatment type, FEV1 percentage, DLCO percentage, PTV volume, mean lung dose; * use proxy SNP in validation; ^ Re-genotyped using original SNP found in discovery phase.

**Table 4:** Unfavorable genotype (UFG) analysis for radiation-induced toxicities.

UFG Group	Number of UFGS	Grade ≥ 2*n* (%)	Grade < 2*n* (%)	Adjusted OR * (95% CI)	*p* Value
**Esophagitis**					
0	0–5	68 (48.92%)	71(51.08%)	1 (reference)	
1	6–7	53 (63.86%)	30 (36.14%)	3.63 (1.56–8.43)	0.003
2	8–10	21 (84.00%)	4 (16.00%)	113.12 (14.73–868.67)	5.46 × 10^−6^
*p* trend for individual UFG				1.78 (1.43–2.22)	2.59× 10^−7^
**Pneumonitis**					
0	0–2	4 (9.76%)	37 (90.24%)	1 (reference)	
1	3–5	72 (40.45%)	106 (59.55%)	8.65 (2.73–27.38)	0.0002
2	6–8	11 (78.57%)	3 (21.43%)	73.05 (11.28–472.94)	6.70 × 10^−6^
*p* trend for individual UFG				2.49(1.79–3.46)	6.34 × 10^−8^

* Adjusted for age, sex, pack year, clinical stage, performance status, concurrent chemoradiotherapy, radiation treatment type, FEV1 percentage, DLCO percentage, PTV volume, mean dose (pneumonitis: mean lung dose; esophagitis: mean esophagus dose and mean lung dose).

**Table 5:** SNP function prediction.

SNP	Related Outcome	Gene	Position	Enhancer Histone Marks	DNase	Promoter Histone Marks	Proteins Bound	Motifs Changed	Amino Acid Change	eQTL
rs7165790	esophagitis	BLM	intronic	-	-	-	-	Barhl1,Zbtb12	-	-
rs8176257	esophagitis	BRCA1	intronic	-	-	-	-	Pax-4,TATA	-	Y
rs2270132	esophagitis	BLM	intronic	HSMM	4 cell types	-	-	Zfp740	-	-
rs12516	esophagitis	BRCA1	3'-UTR	-	-	-	-	9 altered motifs	-	Y
rs1799966	esophagitis	BRCA1	Missense	-	-	-	-	-	S [Ser] ⇒ G [Gly]	Y
rs4873772	esophagitis	PRKDC	intronic	-	-	-	-	CEBPD,EWSR1-FLI1,HDAC2	-	-
rs1822744	esophagitis	TOPBP1	intronic	Huvec	-	-	-	DBP,INSM1,Pax-2	-	-
rs11078671	esophagitis	RPA1	intronic	-	-	-	-	CIZ,E2F	-	-
rs401549	esophagitis	BLM	intronic	HMEC	-	-	-	Foxj1,Pax-3,Sox	-	-
rs1776139	esophagitis	EXO1	intronic	-	-	-	-	4 altered motifs	-	-
rs10514249	esophagitis	XRCC4	intronic	-	WERI-Rb-1	-	-	-	-	-
rs1051772	pneumonitis	TOPBP1	synonymous	-	5 cell types	-	-	21 altered motifs	-	-
rs16944739	pneumonitis	BLM	intronic	K562, GM12878	GM12878, GM12892, GM12864	-	-	TCF12	-	-
rs3735461	pneumonitis	RPA3	intronic	Huvec	18 cell types	-	5 bound proteins	Foxc1,STAT	-	-
rs963248	pneumonitis	XRCC4	intronic	-	-	-	-	VDR	-	-
rs3760412	pneumonitis	EME1	intronic	-	-	-	-	LBP-1,LXR	-	-
rs11571468	pneumonitis	RAD52	intronic	NHEK	Hepatocytes	-	-	-	-	-
rs4986764	pneumonitis	BRIP1	Missense	-	-	-	-	SIX5,THAP1,Znf143	S [Ser] ⇒ P [Pro]	-
rs917029	pneumonitis	EME1	intronic	-	HL-60	-	-	5 altered motifs	-	-

**Table 6:** VEGAS Results for the significant genes for radiation esophagitis and pneumonitis from discovery set.

Gene	Chr	Number of SNPS	*p* Value
**Esophagitis**			
EXO1	1	23	0.005
RPA1	17	16	0.009
MDC1	6	5	0.014
BLM	15	23	0.018
RAD54L	1	18	0.023
BRCA1	17	10	0.024
PRKDC	8	11	0.036
**Pneumonitis**			
RAD54L	1	18	0.033
MUS81	11	6	0.036
RAG1	11	7	0.039
RAG2	11	7	0.039
EME1	17	10	0.048
ATM	11	10	0.049

## Supplementary Materials

Supplementary materials are available online at http://www.mdpi.com/2072-6694/8/2/23/s1.
