# Supplementary material for: Association between Genetic Variants in DNA Double-Strand Break Repair Pathways and Risk of Radiation Therapy-Induced Pneumonitis and Esophagitis in Non-Small Cell Lung Cancer
Source: Cancers (Basel). 2016 Feb 18;8(2):23. doi: 10.3390/cancers8020023 (PMC4773746; doi:10.3390/cancers8020023)
Supplement: Supplementary file 1 [file cancers-08-00023-s001.pdf]

Supplementary Material: Association between Genetic Variants in DNA Double-Strand Break Repair Pathways and Risk of Radiation Therapy-Induced Pneumonitis and Esophagitis in Non-Small Cell Lung Cancer

Lina Zhao, Xia Pu, Yuanqing Ye, Charles Lu, Joe Y. Chang and Xifeng Wu

Table S1. Genes in DSB repair pathway included in this study.

Gene Symbol	Chr	Number of SNPs Included
APTX	9	1
ATM	11	10
BLM	15	23
BRCA1	17	10
BRCA2	13	27
BRIP1	17	13
CHEK1	11	8
CLSPN	1	4
DCLRE1C	10	14
DMC1	22	4
EME1	17	10
EXO1	1	24
LIG1	19	11
LIG3	17	6
LIG4	13	12
MDC1	6	5
MRE11A	11	5
MUS81	11	6
NBN	8	14
PNKP	19	5
PRKDC	8	11
RAD50	5	5
RAD51	15	5
RAD51C	17	6
RAD51L3	17	7
RAD52	12	19
RAD54B	8	7
RAD54L	1	9
RAG1	11	4
RAG2	11	4
RPA1	17	17
RPA2	1	6
RPA3	7	30
RPA4	X	3
SHFM1	7	7
SMC1L1	X	1
SPO11	20	1

Table S1. *Cont.*

Gene Symbol	Chr	Number of SNPs Included
TOPBP1	3	10
TP53BP1	15	6
XLF	2	7
XRCC2	7	10
XRCC3	14	6
XRCC4	5	21
XRCC5	2	25
XRCC6	22	1

Table S2. SNPs in DSB pathway associated with the risk of esophagitis (50 SNPS).

SNP	Gene	Genotype	Chromosome	Model	OR (95% CI)	*p* Value
rs799923	BRCA1	G > A	17	ADD	0.39 (0.22–0.69)	0.001
rs16945643	BRIP1	A > G	17	ADD	0.2 (0.08–0.53)	0.001
rs2797604	EXO1	A > G	1	DOM	0.29 (0.13–0.64)	0.002
rs6413436	RAD52	A > G	12	DOM	0.34 (0.16–0.69)	0.003
rs3786136	RPA1	G > A	17	ADD	2.64 (1.4–4.97)	0.003
rs4149909	EXO1	A > G	1	DOM	0.15 (0.04–0.56)	0.004
rs1592159	DCLRE1C	A > G	10	ADD	0.49 (0.3–0.81)	0.005
rs10744729	RAD52	C > A	12	DOM	0.33 (0.15–0.71)	0.005
rs4149963	EXO1	G > A	1	DOM	6.39 (1.71–23.89)	0.006
rs4873772	PRKDC	G > A	8	REC	7.17 (1.77–29.05)	0.006
rs8178108	PRKDC	A > G	8	DOM	7.03 (1.75–28.27)	0.006
rs2295465	RAD54L	A > C	1	DOM	0.36 (0.18–0.75)	0.007
rs2270132	BLM	A > C	15	DOM	2.59 (1.27–5.26)	0.009
rs16944894	BLM	A > G	15	ADD	0.46 (0.25–0.84)	0.011
rs8176257	BRCA1	C > A	17	REC	5.42 (1.47–20.03)	0.011
rs12118937	EXO1	G > A	1	REC	28.27 (2.11–379.55)	0.012
rs11078671	RPA1	C > A	17	ADD	2 (1.16–3.43)	0.012
rs401549	BLM	A > G	15	ADD	1.91 (1.14–3.2)	0.013
rs3094093	MDC1	T > A	6	DOM	4.49 (1.36–14.86)	0.014
rs11852361	BLM	G > A	15	ADD	0.38 (0.17–0.84)	0.016
rs2046663	RAD54B	G > A	8	DOM	0.31 (0.12–0.81)	0.017
rs2237060	RAD50	A > C	5	REC	3.25 (1.22–8.63)	0.018
rs2230931	RPA1	A > G	17	ADD	2.49 (1.17–5.29)	0.018
rs1799944	BRCA2	A > G	13	DOM	12.1 (1.5–97.4)	0.019
rs2602141	TP53BP1	A > C	15	REC	8.54 (1.43–51.04)	0.019
rs766173	BRCA2	A > C	13	DOM	19.98 (1.59–251.06)	0.020
rs7916726	DCLRE1C	A > G	10	REC	0.22 (0.06–0.8)	0.021
rs1822744	TOPBP1	A > G	3	ADD	1.86 (1.1–3.13)	0.021
rs7310449	RAD52	G > A	12	DOM	0.41 (0.2–0.88)	0.022
rs10514249	XRCC4	A > G	5	REC	0.39 (0.17–0.89)	0.024
rs17761467	RPA1	G > A	17	ADD	2.28 (1.11–4.69)	0.025
rs2298483	CHEK1	G > A	11	ADD	0.45 (0.22–0.91)	0.026
rs9520823	LIG4	A > C	13	ADD	1.85 (1.07–3.21)	0.028
rs7167216	BLM	G > A	15	ADD	0.42 (0.19–0.92)	0.030
rs3212102	XRCC3	G > A	14	DOM	0.2 (0.05–0.85)	0.030
rs12516	BRCA1	G > A	17	REC	3.89 (1.12–13.54)	0.032
rs1799966	BRCA1	A > G	17	REC	3.89 (1.12–13.54)	0.032

Table S2. *Cont.*

SNP	Gene	Genotype	Chromosome	Model	OR (95% CI)	*p* Value
rs11571686	BRCA2	A > C	13	ADD	0.44 (0.21–0.94)	0.033
rs1776179	EXO1	G > A	1	ADD	1.77 (1.05–2.98)	0.033
rs13029742	XLF	C > A	2	DOM	0.48 (0.25–0.94)	0.033
rs4750566	DCLRE1C	C > A	10	ADD	0.57 (0.34–0.96)	0.035
rs7165790	BLM	A > G	15	ADD	0.6 (0.37–0.97)	0.037
rs1776148	EXO1	G > A	1	DOM	2.05 (1.04–4.04)	0.038
rs2301936	RPA3	A > C	7	DOM	0.42 (0.18–0.96)	0.040
rs2073490	EXO1	G > A	1	REC	0.18 (0.03–0.95)	0.043
rs1011980	XRCC4	A > G	5	REC	8.81 (1.07–72.31)	0.043
rs1776139	EXO1	A > C	1	DOM	0.45 (0.21–0.98)	0.044
rs5030740	RPA1	A > G	17	ADD	0.58 (0.34–0.99)	0.047
rs2238337	BLM	G > A	15	DOM	0.49 (0.24–1)	0.049
rs3095329	MDC1	A > G	6	ADD	0.56 (0.32–1)	0.049

* adjusted for age, sex, pack year, clinical stage, performance status, concurrent chemoradiotherapy, radiation treatment type, FEV1 percentage, DLCO percentage, PTV volume, mean esophagus dose and mean lung dose. Abbreviations: Dom, dominant; Rec, recessive; Add: additive.

Table S3. Proxy SNPs used for radiation esophagitis related SNP validation.

SNP	Proxy SNP
rs7165790	rs7175811
rs716579	rs7175811
rs12516	rs8176265
rs4873772	rs7842068
rs1822744	rs1867504
rs11078671	rs2287321
rs177613	rs2526698
rs10514249	rs1011981

Table S4. SNPs in DSB pathway associated with the risk of pneumonitis (31 SNPs).

SNP	Gene	Genotype	Chromosome	Model	OR * (95% CI)	*p* Value
rs1051772	TOPBP1	A > G	3	DOM	0.27 (0.11–0.65)	0.004
rs3735461	RPA3	A > G	7	DOM	2.9 (1.39–6.04)	0.005
rs8178179	PRKDC	A > C	8	ADD	3.68 (1.44–9.39)	0.006
rs3760412	EME1	A > G	17	DOM	0.42 (0.22–0.79)	0.008
rs7503173	RPA1	C > A	17	DOM	2.33 (1.22–4.48)	0.011
rs228606	ATM	C > A	11	DOM	2.32 (1.19–4.53)	0.014
rs1805810	NBN	A > G	8	DOM	0.28 (0.1–0.78)	0.015
rs12283331	RAG2	G > A	11	DOM	2.13 (1.14–3.96)	0.018
rs1818545	RAG2	G > A	11	DOM	2.13 (1.14–3.96)	0.018
rs13817	MUS81	A > G	11	REC	2.86 (1.19–6.89)	0.019
rs558114	MUS81	A > C	11	REC	2.86 (1.19–6.89)	0.019
rs635375	MUS81	A > G	11	REC	2.86 (1.19–6.89)	0.019
rs301275	XRCC4	T > A	5	REC	6.09 (1.34–27.68)	0.019
rs13180316	XRCC4	G > A	5	DOM	0.48 (0.26–0.89)	0.019
rs301289	XRCC4	G > A	5	DOM	2.07 (1.09–3.92)	0.026
rs16944739	BLM	G > A	15	REC	3.11 (1.13–8.56)	0.028
rs10257162	SHFM1	G > A	7	DOM	0.51 (0.28–0.93)	0.029
rs7311263	RAD52	A > C	12	ADD	0.54 (0.3–0.94)	0.030

Table S4. *Cont.*

SNP	Gene	Genotype	Chromosome	Model	OR * (95% CI)	p Value
rs917029	EME1	A > G	17	REC	2.57 (1.09–6.08)	0.031
rs2286028	RAD52	G > C	12	REC	4.86 (1.14–20.64)	0.032
rs4986764	BRIP1	G > A	17	REC	2.42 (1.08–5.45)	0.032
rs13229270	RPA3	C > A	7	DOM	1.97 (1.05–3.71)	0.035
rs17692629	BRCA2	A > G	13	ADD	1.95 (1.05–3.65)	0.036
rs4149963	EXO1	G > A	1	ADD	2.6 (1.06–6.34)	0.036
rs6413436	RAD52	A > G	12	REC	2.44 (1.05–5.69)	0.039
rs11652980	BRIP1	G > C	17	DOM	0.3 (0.09–0.94)	0.040
rs11571468	RAD52	G > A	12	DOM	0.35 (0.13–0.95)	0.040
rs170548	ATM	A > C	11	ADD	1.57 (1.01–2.43)	0.043
rs9350	EXO1	G > A	1	DOM	0.5 (0.25–0.98)	0.044
rs963248	XRCC4	A > G	5	DOM	1.92 (1.01–3.63)	0.047
rs1011980	XRCC4	A > G	5	DOM	0.54 (0.29–1)	0.049

* adjusted for age, sex, pack year, clinical stage, performance status, concurrent chemoradiotherapy, radiation treatment type, FEV1 percentage, DLCO percentage, PTV volume, mean lung dose.

Table S5. Proxy SNPs used for radiation pneumonitis related SNP validation.

SNP	Proxy SNP
rs16944739	rs7403304
rs96324	rs35268
rs3760412	rs12452484
rs4986764	rs6504063
rs917029	rs7211667

Table S6. Distribution of radiation-induced toxicities in patients treated with radiation only and concurrent chemoradiation.

Treatment	Discovery	Validation
Radiation	65 (26)	110 (65)
Esophagitis		
No	55 (85.9)	85 (77.3)
Yes	9 (14.1)	25 (22.7)
Pneumonitis		
No	39 (65.0)	57 (60.6)
Yes	21 (35.0)	37 (39.4)
Concurrent chemoradiation	185 (74)	60 (35)
Esophagitis		
No	51 (27.6)	19 (32.2)
Yes	134 (72.4)	40 (67.8)
	p * < 0.001	p * < 0.001
Pneumonitis		
No	108 (62.4)	34 (61.8)
Yes	65 (37.6)	21 (38.2)
	p * = 0.722	p * = 0.887

* Chi-square comparison of treatment-induced toxicity between radiation-only and concurrent chemoradiation treatment groups in discovery and validation.
